# Three new species of buprestid genus *Aphanisticus* Latreille, 1810 from Southern African countries (Coleoptera, Buprestidae), with nomenclatural and taxonomic notes

**DOI:** 10.3897/zookeys.1287.200030

**Published:** 2026-08-03

**Authors:** Mark Yu. Kalashian, Vítězslav Kubáň

**Affiliations:** 1 Institute of Zoology, Scientific Centre of Zoology and Hydroecology, 7 P. Sevak St., Yerevan 0014, Armenia Institute of Zoology, Scientific Centre of Zoology and Hydroecology Yerevan Armenia; 2 96/43 Hřbitovní, 66451 Šlapanice, Czech Republic Unaffiliated Šlapanice Czech Republic

**Keywords:** Botswana, jewel beetles, lectotype designation, new synonymy, Republic of South Africa, taxonomy, Zambia

## Abstract

Three new species of *Aphanisticus* are described from Southern Africa. *Aphanisticus fencli***sp. nov**., from the Republic of South Africa, belongs to the newly proposed *A. gebhardti* Obenberger, 1924 species group. Additionally, *A. franki***sp. nov**. from Botswana and *Aphanisticus snizeki***sp. nov**. from Zambia are described and compared to related species. The *A. gebhardti* species group includes *A. lineolatus* Obenberger, 1928 and *A. planatus* Théry, 1930. Lectotypes for *A. gebhardti* and *A. lineolatus* are designated and new synonymy, *A. lineolatus* = *A. semicoeruleus*, **syn. nov**., is established. A key to the species of the group is provided.

## Introduction

The jewel-beetle genus *Aphanisticus* Latreille, 1810 is a diverse group of the family Buprestidae (Coleoptera), which includes about 360 species in warm-temperate to tropical zones of the Eastern Hemisphere; of these, 147 species are known from the Afrotropical Region (Bellamy 2008). Thanks to the kindness of our Czech colleagues, we had the opportunity to study numerous and diverse materials of the genus from the region, among which were three remarkable new species. Descriptions of these species are given below. One of the species described belongs to the newly established species-group; taxonomic and nomenclature notes on the species of this group are provided.

## Materials and methods

The present study is based mainly on extensive materials collected in recent years by Czech entomologists. Some materials from other collections were studied as well.

The following abbreviations are used in the text to indicate the depositary of specimens:

**DFPC** David Frank collection, Prague, Czech Republic;

**NHMUK** The Natural History Museum, London, United Kingdom;

**MKCY** Mark Kalashian research collection, Yerevan, Armenia;

**MNHN** Muséum national d’Histoire naturelle, Paris, France;

**NMPC** National Museum, Prague, Czech Republic;

**SAMC** Iziko Museum of Cape Town, Republic of South Africa;

**ZIN** Zoological Institute RAS (Saint Petersburg, Russia).

Specimens were examined using Micromed MC-2 Zoom and MBS-10 stereomicroscopes, and the measurements were made using an ocular micrometer. Photographs were taken using a Canon EOS 550D digital camera equipped with a Canon MP-E65 mm f/2.8 1–5× macro lens. Helicon Focus Pro software was used for focus-stacking photographs.

Label data are cited verbatim; a slash ( / ) is used to separate data from separate labels. The following notations are used: (h) – handwritten, (p) – printed; if necessary, current geographical names and other necessary explanatory information to supplement the data on the labels are given in square brackets ( [ ] ). Authors’ labels with a designation of the type status of the specimens (holotype, lectotype, etc.) are not cited in the text.

## Systematics

### Order Coleoptera Linnaeus, 1758

**Suborder Polyphaga Emery, 1886**

**Superfamily Buprestoidea Leach, 1815**

**Family Buprestidae Leach, 1815**

**Subfamily Agrilinae Laporte, 1835**

**Tribe Aphanisticini Jacquelin du Val, 1863**

**Genus *Aphanisticus* Latreille, 1810**

**Species group *Aphanisticus gebhardti* Obenberger, 1924, gr. nov**.

**Figures 1–8.**
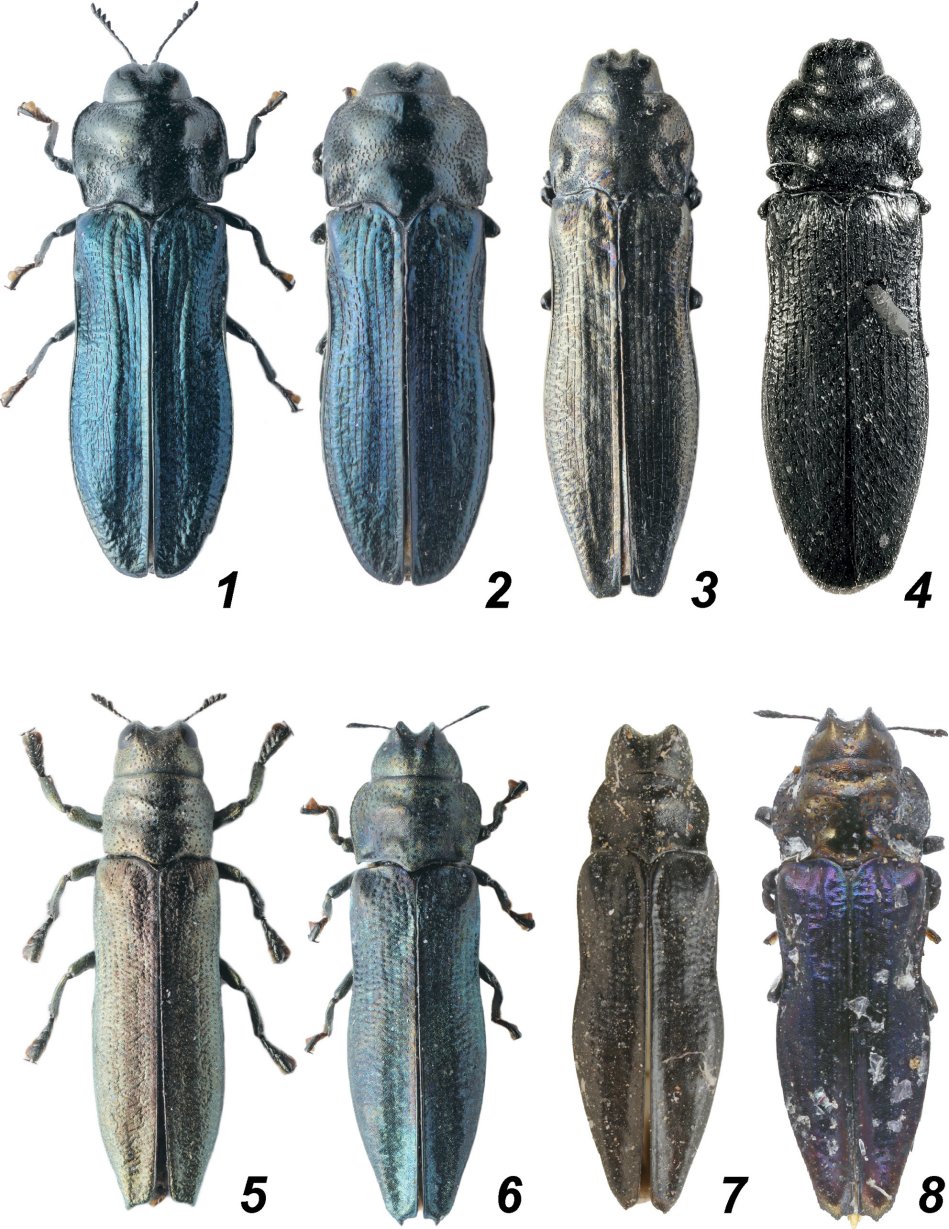
*Aphanisticus* spp., habitus. **1**. *A. fencli* sp. nov. (holotype); **2**. *A. lineolatus* Obenberger, 1928 (lectotype); **3**. *A. gebhardti* Obenberger, 1924 (lectotype); **4**. *A. planatus* Théry, 1930; **5**. *A. franki* sp. nov. (holotype); **6**. *A. snizeki* sp. nov. (holotype); **7**. *A. mitratus* (Chevrolat, 1838), Madagascar; **8**. *A. coeruleipennis*Théry 1930 (holotype).

**Description**. Body (Figs 1–4) large, 3.95–7.0 mm long, elongate, 3.05–3.55 times as long as wide, flattened; head small, distinctly narrower than anterior margin of pronotum, with frontovertex concavity shallow; eyes slightly convex, directed forward. Pronotum moderately transverse, 1.3–1.35 times wider than long, somewhat shield-shaped, with narrowly separated elevations. Elytra with more or less distinct striate structure.

This group can be easily distinguished by a combination of characters unique to the genus *Aphanisticus*. The genus includes morphologically very diverse species and species groups, some of which are established formally (Kalashian 2003, 2004, 2005, 2020; Kalashian and Kubáň 2014). Most species of *Aphanisticus* are characterised by a convex, subcylindrical body, but in several species the body is rather widely oval or subtriangular. The pronotum can be hemispherical or with more or less developed fovea and elevations. Only a few species have subparallel, flattened elytra (species groups *A. diabolicus* Deyrolle, *A. javaecola* Obenberger, 1932 and some species from species group *A. bicolor* Kerremans, 1898; Kalashian 2004); all these species differ by their smaller body, (length does not exceed 3.8 mm) and by more or less pronounced longitudinal costae on the elytra.

Besides *A. gebhardti* Obenberger, 1924, the *A. gebhardti* group includes also *A. lineolatus* Obenberger, 1928, *A. planatus* Théry, 1930, and *A. fencli* sp. nov. decribed below. These species of the group are known so far exclusively from the Republic of South Africa.

#### Aphanisticus fencli sp. nov.

Taxon classificationAnimaliaColeopteraBuprestidae

15746933-C14E-56EE-95E7-D9D66037FEA5

https://zoobank.org/36349957-2C11-486D-9FE4-B810138A2A93

Figs 1, 9

##### Type material.

***Holotype***. Republic Of South Africa • ♂, [KwaZulu-]NATAL prov.; BULWER; 29°48'S, 29°44'E; 11.XI.2001; Dr R. Fencl leg.; NMPC (Fig. 1). ***Paratypes***. • 10 ♂♂; 8 ♀♀; 5 ex. sex indef., same data as for holotype; NMPC; MKCY; ZIN.

##### Description.

***Body*** (Fig. 1) flattened, elongate, 3.05–3.25 times as long as wide, black, with indistinct bluish lustre; elytra blue; surface microreticulated, very delicately on head, pronotum, and most of ventral surface; in elytra and propleurae rather distinct, surface with silky lustre. Length 3.95–5.95 mm (holotype 4.5 mm), width 1.7–1.95 mm (holotype 1.4 mm).

***Head*** rather narrow, with sides somewhat irregularly, arcuately converging toward front. Eyes rather small, slightly convex, directed forward. Oculofrontal board rounded. Frons very slightly widening towards back (to above in frontal view), with deep, doubled postclypeal fovea. Frontovertex shallowly concave; concavity rounded posteriorly, not reaching as far as anterior margin of pronotum. Frons with a few, nearly indistinct traces of small punctures; vertex with irregular, moderately dense, rather large, flat punctures. Antennae serrate from antennomere 8; antennomeres 1 and 2 large, swollen; antennomeres 3–7 rather long and thin, very finely enlarged distally; antennomere 8 nearly equilateral, antennomere 9–11 transversal.

***Pronotum*** 1.35–1.5 times as wide as long, somewhat shield-shaped, widest between anterior and posterior 1/3; sides in anterior 1/4 nearly regularly arcuate, middle 1/3 nearly straight, subparallel, posteriorly sinuate before nearly straight rounded posterior angles; anterior margin slightly bisinuate, posterior margin bisinuate with moderately wide, rounded medial lobe. Lateral margins with traces of very indistinct serration, fringed with continuous, thin groove. Pronotum with shallow elevations: wide, triangular elevation along anterior margin; large transversal elevations near anterior 1/3 and along basal margin, and a couple of longitudinal elevations laterally along basal 1/2. Surface with large, rather dense, flattened, irregular punctures, somewhat smoothed and becoming smaller on elevated portions of disc.

***Scutellum*** very small, nearly equilaterally triangular.

***Elytra*** 2.2–2.4 times as long as wide, slightly widening just behind humery, then sides sinuate approximately to posterior 2/5, where elytra become widest, then finely, convexly narrowed to slightly separately irregularly rounded, very finely serrate apices; sides fringed with thin, distinct groove posteriorly reaching approximately 1/6 of elytral length. Elytra flattened, with distinctly elevated suture and 5^th^ interval; surface between these elevations with thin, in places shortly interrupted, longitudinal striae not reaching elytral apex, laterally of 5^th^ interval with rather tangled, thin, longitudinal hyphens in places fused into short striae; apically elytra with few rather rough irregular punctures and transversal wrinkles.

***Ventral surface***. Prosternal process with a few flat, irregular punctures; proplevrae with single, nearly indistinct traces of small punctures; rest of ventral surface with dense, large, flat, round punctures slightly smoothed towards back.

**Figures 9–11.**
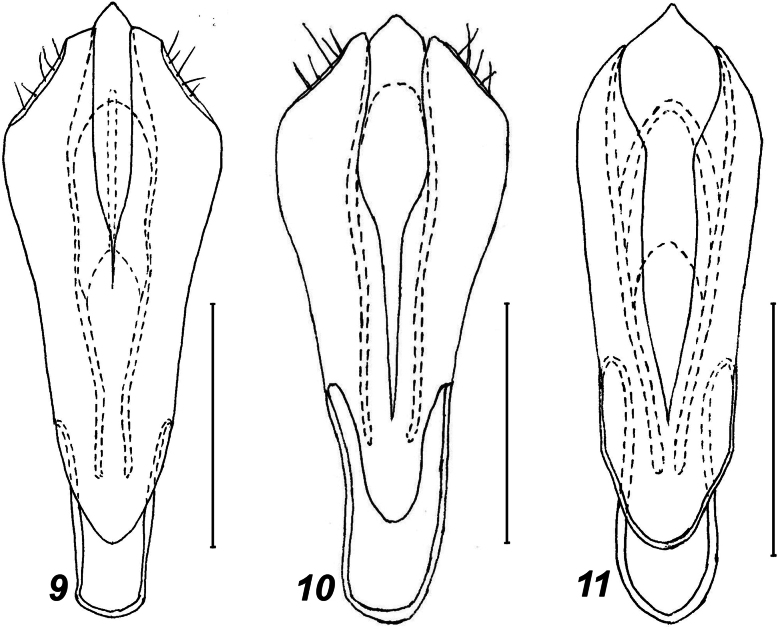
*Aphanisticus* spp., aedeagus. **9**. *A. fencli* sp. nov. (paratype); **10**. *A. lineolatus* Obenberger, 1928 (lectotype); **11**. *A. franki* sp. nov. (paratype). Scale bars: 0.5 mm.

***Aedeagus*** as shown in Fig. 9.

***Sexual dimorphism*** very slightly pronounced; anal ventrite in male shortly nearly straightly cut, in female nearly regularly rounded distally.

##### Differential diagnosis.

See key below.

##### Type locality.

The Republic of South Africa, Natal Province, Bulwer, 29°48'S, 29°44'E.

##### Distribution.

The Republic of South Africa; so far known from type locality only.

##### Etymology.

The new species is named after the blessed memory of JUDr Rudolf Fencl, Czech judge and amateur entomologist, who collected the type series. He was founder and first head of the West Czechian branch of the Czech Entomological Society.

#### Aphanisticus lineolatus

Taxon classificationAnimaliaColeopteraBuprestidae

Obenberger, 1928

3ABA047B-016F-5B8E-8A66-0B3CF2E25D6C

Figs 2, 10

Aphanisticus lineolatus Obenberger, 1928: 78.
Aphanisticus semicoeruleus Théry, 1930: 172, syn. nov.

##### Type material.

*Aphanisticus lineolatus*. ***Lectotype***. Republic Of South Africa • ♂ P. B. Spei; Transvaal (h)/ Fry Coll. 1905.100 (p)/ TYPUS (p, red paper)/ Aphanisticus; lineolatus m.; Type (h); Det Dr. Obenberger (p); NMPC (Fig. 4). ***Paralectotype***. • Sex indef. Cap B. Spei (h)/ TYPUS (p, red paper)/ TYPE (p, red paper)/ Aphanisticus lineolatus m. Type (h); Det Dr. Obenberger (p); MNHN.

*Aphanisticus semicoeruleus*. ***Holotype***. Republic Of South Africa • sex indef. P.B. Spei; Transvaal (h)/ Fry Coll.; 1905.100 (p)/ semicoeruleus Thery (h) TYPE (p, red)/ exp. dessine (h)/ Aphanisticus; lineolatus m.; Type (h); Det Dr. Obenberger (p)/ TYPE (p, red paper)/ APPARTIENT AU; BRITISH MUSEUM INALIENABLE (p, red); MNHN, should be transferred to BMNH.

##### Type locality.

The Republic of South Africa, Cape of Good Hope (according to the lectotype label).

##### Distribution.

According to the primary description, the species was found in a few localities of the Republic of South Africa: “Haec species mihi e locis sequentibus nota est: Transvaal, Ludenburg District (Mus. Capetown), Cap Bonae spei; Ayres; (Coll. Fry in British Museum of Natural History)” (Obenberger 1928).

##### Notes.

It can be assumed that Théry (1930) described, for unclear reasons, *A. semicoeruleus* from one of the specimens in the type series of Obenberger’s *A. lineolatus*. A comparison of the lectotype of *A. lineolatus* with the holotype of *A. semicoeruleus* showed their conspecifity. According to the primary description, one additional paralectotype is deposited in SAMC; this specimen was mentioned by Bellamy (2008), but its whereabouts is unknown to us.

#### Aphanisticus gebhardti

Taxon classificationAnimaliaColeopteraBuprestidae

Obenberger, 1924

06F299D3-48A3-561B-88F7-E81D1035DD67

Fig. 3

Aphanisticus gebhardti Obenberger, 1924: 158.

##### Type material.

***Lectotype***. Republic Of South Africa • ♀, Kapstadt (p); Nov. 1922 (h) / TYPUS (p, red paper) / auf *Watsonia rosea*; fressend (h) / auf *Watsonia rosea* (h) / Aphanisticus gebhardti m.; Type (h); Det. Dr. Obenberger (p); NMPC.

##### Material examined.

Republic Of South Africa • 1 ex., Tafelberg; Capstadt (h) / 14.X.23 (h); Dr. Brauns (p) / Aphanisticus gebhardti (h, pencil script) / Cum TYPO comparavit; dr. v. Gebhardt (p, yellow paper) / Typus (p, red paper)/ question mark (p, red paper) / coll. THERY (p); MNHN • 1 ex.: Tafelberg; Capstadt (h) / auf *Watsonia rosea* (h) / 14.X.23 (h); Dr. Brauns (p) / Aphanisticus (p); gebhardti Obb. (h); Thery det (p) / exemplaire dessine (p) / Collect. THERY (p) / red square/ Compar. a paratype; par A. Thery (h); MNHN.

##### Type locality.

The Republic of South Africa, Cape town.

##### Distribution.

So far, known from the type locality only.

##### Biological notes.

According to the description and label data, this species was collected in November on *Watsonia rosea* KerGawl. (currently synonym of *Watsonia borbonica* (Pourr.) Goldblatt).

#### Aphanisticus planatus

Taxon classificationAnimaliaColeopteraBuprestidae

Théry, 1930

BEC421C8-AC36-5E14-93C3-59BF26282A4D

Fig. 4

Aphanisticus planatus Théry, 1930: 184.

##### Type material.

***Holotype***. Republic Of South Africa • sex indef., Cap (h) / Cap. B. E. (h); Coll. Van de Pool (p) / Aphanisticus planatus Thery (h); Type (p, red) / Exp. dessine (h) / TYPE (p. red paper); MNHN.

##### Type locality.

The Republic of South Africa, Cape of Good Hope.

##### Distribution.

So far, known from the type locality only.

### Key to the species of *A. gebhardti* Obenberger, 1924 species group

**Table:** 

1	Dorsal surface bicolour; pronotum black, with indistinct bluish lustre, and elytra blue. Body less elongated, 3.05–3.25 times as long as wide; oculofrontal borders rounded	**2**
–	Dorsal surface unicolour, bronzy, or blackish bronze. Body more elongate, > 3.3 times as long as wide; oculofrontal borders form projection along inner margins of eyes	**3**
2	Sides of pronotum less convergent towards back, with lateral fringe pronounced only near posterior angles; punctuation on pronotum deeper and smaller; striate structure of elytra more regular; body 5.2–5.4 mm long	***A. lineolatus* (Fig. 2)**
–	Sides of pronotum more distinctly convergent towards back, fringed with continuous, thin groove; punctuation of pronotum shallower and somewhat larger; striate structure of elytra less regular, with striae in places shortly interrupted; body 3.95–5.95 mm long	***A. fencli* sp. nov. (Fig. 1)**
3	Dorsal surface bronzy, with olivaceous reflection; body more elongated, 3.5 times as long as wide; elytra 2.6 times as long as wide; projection along inner margins of eyes more pronounced; body 6.1–7.0 mm long	***A. gebhardti* (Fig. 3)**
–	Dorsal surface blackish bronze with indistinct, olivaceous reflection; body less elongated, 3.3 times as long as wide; elytra 2.5 times as long as wide; projection along inner margins of eyes less pronounced; body 6.0 mm long	***A. planatus* (Fig. 4)**

#### Aphanisticus franki sp. nov.

Taxon classificationAnimaliaColeopteraBuprestidae

78C41012-3FB5-5FED-BA75-E1E11F129695

https://zoobank.org/41F89DA3-1D46-40CC-AE8A-EE3D1348382A

Figs 5, 11

##### Type material.

***Holotype***. Botswana • ♂, West Okawango South Delta; 17.XI.2000; Lgt. P. Macháček.; NMPC (Fig. 5). ***Paratypes***. • 8 ♂♂; 6 ♀♀; 5 ex. sex indef., same data as for holotype; NMPC; DFPC; MKCY.

##### Description.

***Body*** (Fig. 5) convex, subcylindrical, elongate, 3.55–3.75 times as long as wide, olivaceous-green, in places with indistinct bronzy reflection; surface distinctly microreticulated, with silky lustre; length 4.7–5.5 mm (holotype 4.8 mm), width 1.3–1.5 mm (holotype 1.3 mm).

***Head*** large, subequal in width to anterior margin of pronotum, with sides widely, arcuately, slightly converging towards front. Eyes large, convex, well visible from above. Oculofrontal board shortly rounded, forming obtuse keel. Frons very slightly, sinuately, distinctly narrowed towards back, with deep, doubled postclypeal fovea and triangular depression behind them reaching approximately level of anterior 1/3 of eyes. Frontovertex concave; concavity rounded posteriorly, not reaching as far as anterior margin of pronotum. Anterior surface of frons with a few, nearly indistinct traces of small punctures; posterior concavity and vertex with moderately dense, small, rather deep, irregular punctures. Antennae serrate from antennomere 8; antennomeres 1 and 2 large, swollen; antennomeres 3–7 short; antennomeres 3–5 very finely and 6–7 distinctly enlarged distally; antennomeres 8–11 strongly transversal.

***Pronotum*** 1.15–1.20 times as wide as long, widest before middle, sides anteriorly slightly arcuately convergent toward anterior angles, posteriorly very slightly and nearly straight convergent toward approximately straight posterior angles; anterior margin very slightly bisinuate and posterior margin bisinuate with broad, rounded medial lobe. Lateral margins very indistinctly, irregularly serrate, fringed with indistinct, thin grove. Pronotum nearly regularly convex; disc with two slightly separated transversal elevations near anterior 1/3 and in front of basal margin. Punctuation of pronotum similar to that of vertex.

***Scutellum*** small, transversely triangular.

***Elytra*** 2.5–2.65 times as long as wide, slightly widening just behind humery, then sides feebly sinuate approximately to posterior 2/5, where elytra become widest, then finely convexly, slightly sinuately narrowed distally; each elytron apically prolonged to distinct lateral tooth. Elytra convex, slightly depressed along posterior half of suture. Surface with rows of small rounded punctures smoothed posteriorly, and with few thin irregular wrinkles in posterior 2/5–1/3.

***Ventral surface***. Prosternal process very feebly depressed medially, without punctuation, the rest of surface with rather dense, irregular punctures, large and rough anteriorly, on sternum, and base of abdomen; punctures smoothed and become smaller toward back and hardy visible in anal ventrite.

***Aedeagus*** as shown in Fig. 11.

***Sexual dimorphism*** slightly pronounced; anal ventrite widely cut distally in both sexes: in male with small medial incision, in female without incision.

##### Differential diagnosis.

This peculiar species shows somewhat superficial resemblance to *A. mitratus* (Chevrolat, 1838) (Fig. 7) from Madagascar, which can be easily distinguished by its bronzy-black body, more robust than in *A. franki* sp. nov.; *A. mitratus* is approximately 3.2 times as long as wide; the pronotum is 1.3 times as wide as long, and the elytra 2.35 is times as long as wide. Additionally, *A. mitratus* is characterised by having less distinct microreticulation than in the new species; the sides of the pronotum are widely arcuate in the anterior 2/3 and distinctly sinuate before sharp posterior angles, and elevations on the disc are more pronounced; the apical teeth of the elytra are much shorter than in the new species.

##### Type locality.

Botswana, south delta of the West Okawango River.

##### Distribution.

Botswana, so far known from the type locality only.

##### Etymology.

The new species is dedicated, with our gratitude and respect, to well-known specialist on Buprestidae, David Frank, who provided us with the material on the new species.

#### Aphanisticus snizeki sp. nov.

Taxon classificationAnimaliaColeopteraBuprestidae

6129D20A-833F-51C9-81DC-36951361C9C0

https://zoobank.org/A4108D37-3AC5-4A39-9EA6-A5F736A0A350

Fig. 6

##### Type material.

***Holotype***. Zambia • ♀, Kapiri Mposhi; (100–140 km NE); ATB Lodge env.; 15.XII.2004; M. Snižek, V. Tichý leg.; NMPC (Fig. 3).

##### Description.

***Body*** (Fig. 6) convex, subcylindrical, elongated, 3.6 times as long as wide, dorsally bluish-green with indistinct violet reflection, ventrally black with indistinct bronzy lustre; surface microreticulated; dorsal surface and pronotum with silky lustre; reticulation ventrally smoothed towards back and hardly visible on abdominal ventrites. Length 5.05 mm, width 1.4 mm.

***Head*** large, slightly narrower than anterior margin of pronotum, with sides irregularly, arcuately, strongly convergent frontwards. Eyes large, directed sideways, moderately convex, visible from above by small portion. Oculofrontal board sharp, keeled. Frons distinctly, somewhat sinuately widening towards back (to above in frontal view), with deep, doubled postclypeal fovea and triangular depression behind them reaching approximately level of anterior 1/3 of eyes. Frontovertex deeply concave; concavity posteriorly narrowed, nearly reaching anterior margin of pronotum. Anterior surface of frons nearly glabrous, posterior with concavity, and vertex with small, sparse, irregular punctures. Antennae serrate from antennomere 8. Antennomeres 1 and 2 large, swollen; antennomeres 3–7 moderately long, thin, very finely, distally enlarged; antennomere 8 nearly equilateral; antennomeres 9–11 strongly transversal.

***Pronotum*** 1.35 times as wide as long, widely cordiform, widest at approximate middle; sides anteriorly nearly regularly arcuate, slightly sinuate before sharp posterior angles; anterior margin very slightly bisinuate; posterior margin bisinuate, with moderately wide, triangular medial lobe. Lateral margins nearly indistinctly, irregularly serrate, fringed with continuous thin groove. Pronotum narrowly flattened along sides, slightly depressed medially of posterior angles; anterior and posterior margins moderately elevated; disc with slightly separated elevations: pair of transversal elevations near anterior 1/3 and pair of smaller slightly transverse elevations in front of posterior margin. Surface with sparse, irregular, flat punctures of moderate size.

***Scutellum*** very small, nearly equilaterally triangular.

***Elytra*** 2.55 times as long as wide, slightly widening just behind humery, then sides sinuate to posterior approximate 2/5, where elytra become widest, then finely convexly; elytra distally slightly sinuately narrowed to irregularly separately arcuate; apex of each elytron with sharp, triangular medial tooth. Elytra convex, distinctly depressed along elevated suture in posterior 1/2. Surface with rows of small punctures, rather distinct in anterior 3/5, in places with indistinct, irregular, transversal wrinkles; towards back this structure smoothed, apically nearly indistinct.

***Ventral surface***. Prosternal process slightly convex, with few irregular, moderately large, deep punctures; proplevrae without punctuation; sternum with rather large, sparse, irregular, somewhat larger and deeper punctures than in prosternal process; abdomen with small, sparse punctures; abdomen towards back nearly completely smooth. Anal ventrite slightly irregularly arcuate apically.

**Male** unknown.

##### Differential diagnosis.

*Aphanisticus snizeki* sp. nov. is similar to *A. coeruleipennis* Théry, 1930 (Fig. 8), also from Southern Africa, which can be distinguished by smaller, less convex, and more robust body (in *A. coeruleipennis* body 3.15 mm in length, approximately 3.3 times as long as wide; elytra 2.4 times as long as wide). The dorsal surface in *A. coeruleipennis* is somewhat bicolourous with a dark-reddish-violet pronotum and blue-violet elytra with less distinct microreticulation.

##### Type locality.

Zambia, Central Province, Kapiri Mposhi town.

##### Distribution.

Zambia; so far known from the type locality only.

##### Etymology.

The new species is dedicated to one of the collectors of the type series, Miroslav Snížek, with gratitude and respect.

## Supplementary Material

XML Treatment for Aphanisticus fencli

XML Treatment for Aphanisticus lineolatus

XML Treatment for Aphanisticus gebhardti

XML Treatment for Aphanisticus planatus

XML Treatment for Aphanisticus franki

XML Treatment for Aphanisticus snizeki

## Additional information

### Artificial Intelligence (AI) use

The authors accept full responsibility for the content of the manuscript, including the disclosure of any use of AI.

No AI tools were used in the preparation of this manuscript.

### Author ORCIDs

M.Yu. Kalashian https://orcid.org/0000-0002-2448-9547

V. Kubáň https://orcid.org/0009-0004-0598-6785

### Data availability

All of the data that support the findings of this study are available in the main text.
